# The Combined Effect of High-Intensity Interval Training and Time-Restricted Feeding on the AKT-IGF-1-mTOR Signaling Pathway in the Muscle Tissue of Type 2 Diabetic Rats

**DOI:** 10.3390/nu17091404

**Published:** 2025-04-22

**Authors:** Motahareh Mohebinejad, Fatemeh Kazeminasab, Mahtab Ghanbari Rad, Reza Bagheri, Mazdak Razi, Darryn Willoughby, Fred Dutheil

**Affiliations:** 1 Department of Physical Education and Sports Science, Faculty of Humanities, University of Kashan, Kashan 87317-53153, Iran; motaharem1401@yahoo.com; 2 Gerash Cellular and Molecular Research Center, Gerash University of Medical Sciences, Gerash 58666-74417, Iran; m.rad2325@yahoo.com; 3 Department of Exercise Physiology, University of Isfahan, Isfahan 81746-73441, Iran; will.fivb@yahoo.com; 4 Division of Comparative Histology and Embryology, Department of Basic Sciences, Faculty of Veterinary Medicine, Urmia University, Urmia 57561-51818, Iran; mazdak.razi@gmail.com; 5 Department of Education, Innovation, and Technology, Baylor College of Medicine-School of Medicine, Temple, TX 76513, USA; darryn.willoughby@bcm.edu; 6 Preventive and Occupational Medicine, Université Clermont Auvergne, CNRS, LaPSCo, Physiological and Psychosocial Stress, CHU Clermont-Ferrand, University Hospital of Clermont-Ferrand, Witty Fit, F-63000 Clermont-Ferrand, France; fred_dutheil@yahoo.fr

**Keywords:** diabetes, insulin resistance, intermittent fasting, high-intensity interval training, high-fat diet

## Abstract

***Background/Objectives:*** High-intensity interval training (HIIT) and time-restricted feeding (TRF) have shown potential in enhancing glucose metabolism, increasing insulin sensitivity, and promoting muscle health. This study investigates the combined effects of HIIT and TRF on the AKT-IGF-1-mTOR signaling pathway in the muscle tissue of type 2 diabetic (T2D) rats. ***Methods:*** 42 male Wistar rats (4–5 weeks of age) were included in the study. The animals were randomly divided into two groups: 1. Standard diet (SD) non-diabetic (*n* = 7) and 2. High-fat diet (HFD *n* = 35) for 4 weeks. T2D was induced by intraperitoneal injection (IP) of streptozotocin (STZ) at 35 mg/kg. Animals with blood glucose levels ≥ 250 mg/dL were considered diabetic. Diabetic rats were randomly divided into five groups (*n* = 7): 1. Diabetes-HIIT (D-HIIT), 2. Diabetes-TRF (D-T), 3. Diabetes-combined TRF and HIIT (D-T+HIIT), 4. Diabetes-Untreated Control (D), and 5. Diabetes with metformin (D-MET). The HIIT protocol and TRF regimen were followed for 10 weeks. Muscle tissue was collected for histological analysis, and the expression of proteins related to the AKT-IGF-1-mTOR pathway was measured. ***Results:*** Blood glucose levels, insulin resistance (IR), and markers of muscle degradation were significantly improved in the D-T+HIIT and D-MET groups compared to the non-diabetes group. Furthermore, the activation of the AKT and mTOR signaling proteins, as well as increased IGF-1 expression, was significantly elevated in the D-T+HIIT group compared to the diabetic control group and other treatment groups, and approached levels observed in the non-diabetes group. Additionally, muscle fiber size and overall tissue structure were improved in the treatment groups, particularly in the D-T+HIIT group. ***Conclusions:*** The combination of HIIT and TRF appears to offer superior benefits in improving muscle protein synthesis, and glucose regulation in T2D rats, as compared to either HIIT or TRF alone. These findings highlight the potential of this combined approach for addressing muscle-related complications in T2D.

## 1. Introduction

Type 2 diabetes (T2D) is a condition characterized by dysregulated glucose homeostasis, impaired insulin secretion or insulin resistance (IR) [1], which leads to elevated blood glucose levels [2]. In healthy individuals, skeletal muscle absorbs glucose through an insulin-dependent process, but with IR, the muscle’s ability to take up glucose is decreased due to intracellular lipid accumulation, leading to decreased glucose transport and glycogen production [3]. In IR states, the ability of insulin to stimulate glucose uptake in skeletal muscle is significantly impaired [4].

Muscle atrophy is a complication of diabetes that negatively impacts the quality of life through disrupting muscular metabolic interactions and growth [5]. In line with this issue, the AKT–insulin-like growth factor-1 (IGF-1)–mammalian target of rapamycin (mTOR) signaling pathway plays a key role in muscle metabolism, growth, and insulin sensitivity [6]. Indeed, AKT interacts as a key regulator of cellular metabolism and insulin action. In response to IGF-1 (a hormone promoting growth signaling), AKT is activated and facilitates glucose uptake in skeletal muscle. IGF-1 acts similarly to insulin and promotes muscle growth by stimulating mTOR, and consequently regulates protein synthesis, muscle growth, and autophagy [7]. Disruption of this pathway is common in T2D. In particular, IR often leads to impaired AKT activation, decreased glucose uptake, and increased muscle breakdown. Furthermore, reduced mTOR activation can contribute to muscle atrophy and decreased protein synthesis, exacerbating IR [7]. In addition, AKT maintains the protein synthesis process through mTOR and/or protein degradation through fork head box O (FOXO) family transcription factors [8]. Therefore, considering these findings, AKT–IGF-1–mTOR signaling regulates muscle metabolism, growth, and insulin sensitivity. Thus, understanding these mechanisms underscores the importance of targeting this pathway to develop strategies for preserving muscular physiological function and improving metabolic interactions in individuals with T2D.

Any changes in lifestyle, including diet and exercise, are crucial to maintain blood glucose and prevent diabetes-related complications [9]. Intensive lifestyle interventions that combine calorie reduction and increased physical activity with an emphasis on weight loss can improve diabetes symptoms [10]. In line, exercise with different intensities, including aerobic, resistance, and high-intensity interval training (HIIT), have been shown to regulate glucose homeostasis [11]. Among non-pharmacological medical interventions, HIIT and time-restricted feeding (TRF) have gained prominence for their potential to improve metabolic dysregulation in T2D conditions [12,13,14]. HIIT involves short periods of intense exercise followed by periods of low-intensity activity or rest. It activates various signaling pathways, including AMP-activated protein kinase (AMPK) and mTOR-related signaling pathways, both of which play a pivotal role in regulating cellular energy and muscle hypertrophy [15]. As aforementioned, the mTOR pathway is particularly important for protein synthesis, and its activation supports muscle growth and repair. Additionally, the HIIT increases glucose uptake in muscle cells, primarily through the AKT/PKB pathway, which is central to insulin signaling [16,17]. Considering the effectiveness of non-medical interventions in regulating metabolic interactions within muscle cells, investigating their impact on AMP and mTOR expression has gained significant attention in the context of T2D.

In addition to non-medical and medical therapeutic approaches, the TRF involves alternating cycles of eating and fasting [18]. A specific form of intermittent fasting, known as TRF, involves eating with a certain window (e.g., 3 to 12 h) and fasting during the remaining hours of the day. This approach has been shown to improve metabolic health by reducing IR, promoting fat oxidation, and stimulating autophagy [19]. One proposed mechanism underlying TRF’s metabolic benefits is its ability to enhance the IGF-1 signaling pathway [20,21].

Although HIIT and TRF are known to be effective in improving metabolic status and reducing complications of diabetes, the combined effects of these two methods on molecular signaling pathways such as AKT-IGF-1-mTOR, which play a critical role in regulating muscle growth and metabolism, have not yet been fully investigated. Therefore, the aim of the current study was to explore the combined effects of HIIT and TRF on the AKT-IGF-1-mTOR signaling pathway in the muscle tissue of T2D rats by investigating the expression levels of these proteins along with relevant histopathological and biochemical analyses. We utilized this animal model in our study due to its physiological and genetic similarities, ability for better nutrition control, access to tissues for more precise experiments, study of molecular signaling and genetic changes, and investigation of combined effects of treatments.

## 2. Materials and Methods

### 2.1. Animals

This study was conducted in accordance with the ARRIVE guidelines and received approval from the Institutional Review Committee (IR.KASHANU.REC.1402.021) at the University of Kashan. The Code of Ethics was approved on 26 September 2023. All experimental protocols followed the relevant ethical guidelines and regulations. A total of 42 male Wistar rats, aged 4–5 weeks and weighing 200 ± 20 g, were used in this study. The animals were housed in a temperature-controlled environment set at 22 ± 2 °C with a relative humidity of 50 ± 5%, under a 12 h light/12 h dark cycle. At the start of the project, they were given free access to water and a standard pellet diet throughout the study.

### 2.2. Diabetes Induction

After acclimating the rats to their new environment, the rats were randomly divided into two initial groups:

1. Standard diet (SD) (*n* = 7): non-diabetes

2. High-fat diet (HFD) (*n* = 35).

The SD group served as the non-diabetic control, receiving no intervention, while the HFD group was fed a diet consisting of 60% kcals from fat, 20% kcals from carbohydrates, and 20% kcals from protein for 4 weeks to induce T2D (Table 1) [22].

**Figure 1:**
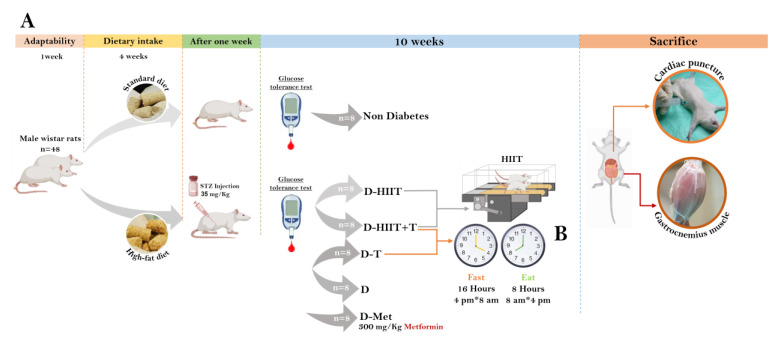
Experimental design. (**A**) A total of 42 male Wistar rats were randomly allocated into two groups: standard diet (SD) and high-fat diet (HFD). After 4 weeks, rats in the HFD group were injected with STZ. The rats with FBG > 250 mg/dL were randomly divided into diabetic groups. After 10 weeks of intervention, the muscle tissue of the rats was extracted. (**B**) Diagram representing the availability of macronutrients during the light and dark periods for each group. Zeitgeber time 0 was set as the beginning of the light period. The following groups had ad libitum access to food and water without time limits: diabetes–HIIT (D-HIIT), diabetes-untreated control (D), and diabetes–MET (D-MET). AL, ad libitum. Blood glucose monitored throughout the treatment of standard and HFD groups.

At the end of the 4-week period, diabetes induction was achieved by intraperitoneal (IP) injection of streptozotocin (STZ) at 35 mg/kg, following 5 h of fasting [23,24]. Diabetes was confirmed via glucose tolerance tests (GTTs), with fasting blood glucose (FBG) levels exceeding 250 mg/dL [25]. After allowing a one-week stabilizing period, during which the rats continued on the HFD, the diabetic rats were randomly assigned to five experimental groups (*n* = 7 per group):

1. Diabetes–HIIT (D-HIIT): Diabetic rats subjected to high-intensity interval training alone.

2. Diabetes–time-restricted feeding (D-T): Diabetic rats undergoing time-restricted feeding alone.

3. Diabetes–TRF and HIIT combined (D-T+HIIT): Diabetic rats receiving both TRF and HIIT interventions.

4. Diabetes–Untreated Control (D): Diabetic rats without intervention, serving as the negative control group.

5. Diabetes–Metformin (D-MET): Diabetic rats treated with metformin, serving as the positive control. The study design is shown in Figure 1.

### 2.3. Processes and Protocols

#### 2.3.1. Adaptation Process

Following T2D induction, the D-HIIT group and the D-T+HIIT groups underwent an adaptation phase to acclimate to treadmill use before the 10-week training period. To ensure the rats could perform at their maximum capacity in the maximum speed test (Vmax), they were first familiarized with the treadmill. During the first week, each rat was placed on the treadmill for 15 min per day at an intensity of 10–15 m/min with zero inclination [26]. To determine Vmax, the rats completed a 5 min warm-up at a speed of 6–10 m/min. Subsequently, the treadmill speed was increased by 3 m/min every 3 min, continuing until the rats exhibited stagnation [27].

#### 2.3.2. The HIIT Protocol

The HIIT protocol was conducted for the D-HIIT and D-T+HIIT groups based on their Vmax test results. The training program lasted 10 weeks, with five sessions per eek. Each session consisted of eight training sets, each lasting three minutes, at an intensity of 85–95% Vo_2_max, followed by a 2 min active rest period at 30–40% Vo_2_max. Additionally, each session began and ended with a 5 min warm-up and cool-down at 30–40% Vmax [28] (Table 2).

#### 2.3.3. IF Protocol

The rats in the D-T and D-T+HIIT groups followed an TRF regimen, consisting of an 8 h feeding period (8:00 a.m. to 4:00 p.m.) with 16 h of fasting (4:00 p.m. to 8:00 a.m.) [29]. The feeding phase began 1 h after the onset of darkness in the animal room. In the current research, the animal room was darkened at 7:00 a.m., the food was provided at 8:00 a.m., and was removed at 4:00 p.m. The lighting was turned on at 7:00 p.m.

### 2.4. Intraperitoneal Glucose Tolerance Test (IPGTT)

To evaluate the impact of HIIT and TRF on glucose metabolism in T2D-induced rats, an intraperitoneal glucose tolerance test (IPGTT) was performed. In line, rats were fasted for 12 h before receiving an IP injection of 2 g/kg body weight of a 20% glucose solution. Blood samples were collected from the tail vein at baseline (immediately before injection), and at 30-, 60-, and 120-min post-injection to measure blood glucose levels.

Blood glucose concentration was determined using the glucose oxidase method with a Glucose-B test kit (Wako, Osaka, Japan) and Accu-Chek Instant S Blood Glucose Glucometer Kit. The area under the curve (AUC) was calculated to access the glucose tolerance and insulin sensitivity. A reduction in AUC in the D-HIIT, D-T, and D-T+HIIT groups indicates improved glucose metabolism and insulin sensitivity compared to the diabetic control group (D) [30].

### 2.5. Muscle Tissue Preparation

To investigate the effects of HIIT and TRF on the AKT-IGF-1-mTOR signaling pathway in the muscle tissue of diabetic rats, gastrocnemius muscle samples were collected after the final intervention. The rats were anesthetized via IP injection of ketamine (50 mg/kg) and Xylazine (10 mg/kg), after which muscle tissue was quickly excised, frozen in liquid nitrogen, and stored at −80 °C. Protein was extracted using a cold lysis buffer and quantified using a Bicinchoninic acid assay (BCA) protein assay. Western blotting was performed to assess the expression and phosphorylation of key signaling proteins involved in the pathway, including AKT, IGF-1, and mTOR.

### 2.6. Western Blot

For protein extraction, muscle tissue was homogenized radioimmunoprecipitation assay (RIPA) lysis buffer, which contains tris-HCl, NaCl, detergents, protease and phosphatase inhibitors, and EDTA or EGTA to ensure efficient cell membrane disruption and protein denaturation. The homogenate was centrifuged at 12,000 rpm, 4 °C for 15 min to remove cellular debris, and the total protein concentration in the supernatant was quantified using the Lowry method [31]. The protein samples were mixed with loading buffer, heated at 95 °C for 5 min, and separated by SDS-PAGE with 10% acrylamide at 120 V. Next, the proteins were transferred to a nitrocellulose membrane at 100 V for 1–2 h, followed by washing in Tris-buffered saline (pH 7.2) with 0.1% Tween 20 and blocking with a 5% milk and 0.5% BSA solution for 1 h at room temperature and overnight at 4 °C. The membranes were incubated with primary antibodies targeting key proteins involved in the AKT-IGF-1-mTOR signaling pathway, including AKT (Elabscience, E-AB-63467, Dilution: 1:600, Houston, TX, USA), IGF-1 (Elabscience, E-AB-70301, Dilution: 1:1000), and mTOR (Elabscience, E-AB-70304, Dilution: 1:500), followed by incubation with a secondary antibody. Protein bands were detected using the Clarity Western ECL Substrate chemiluminescence kit visualized by Laser and CCD-based imaging system (Azure biosystem, AZ600-01, Dublin, CA, USA), and the intensity of the bands was analyzed using an amplified laser densitometer with onboard software [32].

### 2.7. Tissue Sectioning and Staining

The muscles were dissected and fixed in 10% formalin for one week. Subsequently, the tissues underwent a routine dehydration process using increasing concentrations of ethanol (70%, 80%, 90%, 96%, 100%), followed by paraffin embedding. Then, 5–6 μm sections were prepared using a fully automated digital microtome (LKB-Produkter AB, Bromma, Sweden) and stained with Hematoxylin and Eosin (H&E) and Periodic Acid Schiff (PAS) staining dyes [33,34].

### 2.8. Frozen Section Preparation and Sudan Black-B Staining

For frozen section preparation, the muscle tissue samples were rapidly frozen in liquid nitrogen and then stored at −80 °C until further processing. The frozen samples were sectioned into 10–14 μm slices using a cryostat (Leica Biosystems, Nussloch, Germany). The sections were placed on glass slides and air-dried at room temperature for 10 min. For Sudan Black-B staining, the sections were immersed in 0.1% Sudan Black-B solution in isopropanol for 20 min to stain lipid-rich structures. After staining, the sections were washed in 70% ethanol and counterstained with nuclear red dye to nuclei. Finally, the stained sections were mounted with a cover slip using DPX mounting medium and examined under a light microscope (NEXCOPE, NE620, Ningbo, China) for lipid accumulation in the muscle tissue [35].

### 2.9. Biochemical Parameters

FBG and insulin concentrations were measured using standard biochemical techniques. FBG was determined via spectrophotometry with a commercially available glucose kit, and insulin levels were measured using Enzyme-Linked Immunosorbent Assay (ELISA). Insulin sensitivity (IS) was assessed using a key index: The Homeostasis Model Assessment for Insulin Resistance (HOMA-IR). These biochemical assessments provided insight into the metabolic changes following HIIT and TRF interventions in T2D rats. The formula for HOMA-IR is as follows:

HOMA-IR = fasting insulin (microU/L) × fasting glucose (nmol/L)/22.5 [36].
Insulin sensitivity = 1/[log fasting serum insulin (μU/mL) + log fasting plasma glucose (mg/dL)] [37]

### 2.10. Statistical Analysis

Data are presented as means ± standard deviations (SD). Statistical analysis was performed using IBM SPSS Statistics version 26 (IBM Corp., Armonk, NY, USA). A two-way analysis of variance (ANOVA) was used to analyze the HIIT, TRF, metformin, and the interactions between HIIT and TRF (D-T+HIIT). Post hoc comparisons using Tukey’s HSD test were conducted following significant main effects in the two-way ANOVA to determine pairwise differences between groups. A significance level of *p* < 0.05 was considered statistically significant for all analyses.

## 3. Results

### 3.1. Intraperitoneal Glucose Tolerance Tests (IPGTT) in the Experimental Groups

**Figure 2:**
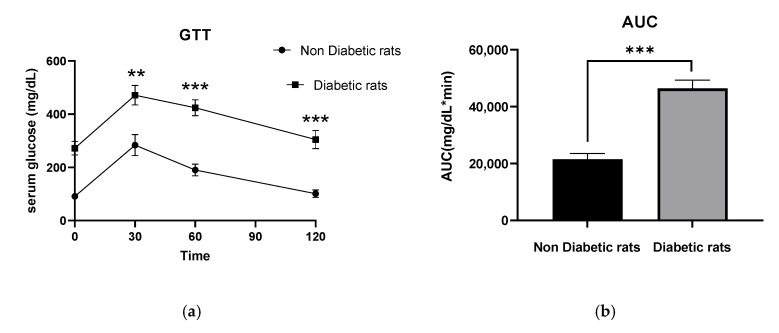
Blood glucose monitored throughout the treatment of standard and HFD groups. (**a**) Intraperitoneal glucose tolerance test (IPGTT) in standard diet and HFD rats after STZ injection and before the start of treatment for the groups (**b**) Total area under the curve (AUC) for the IPGTT was significantly higher in the D rats group compared with the non-diabetic rats. ** *p* < 0.01 and *** *p* < 0.001, compared to non-diabetic rats. All data are shown as mean ± SD.

The IPGTT tests were performed for the non-diabetes and D groups (after STZ injection and before the start of treatment for the groups). IPGTT results, and the corresponding areas under the curve (AUC) for all study groups are depicted in (Figure 2). Blood glucose levels in the D group were increased significantly compared to the non-diabetes group (*p* < 0.001), confirming T2D induction (Figure 2).

### 3.2. Serum Blood Glucose, Insulin, HOMA-IR

**Figure 3:**
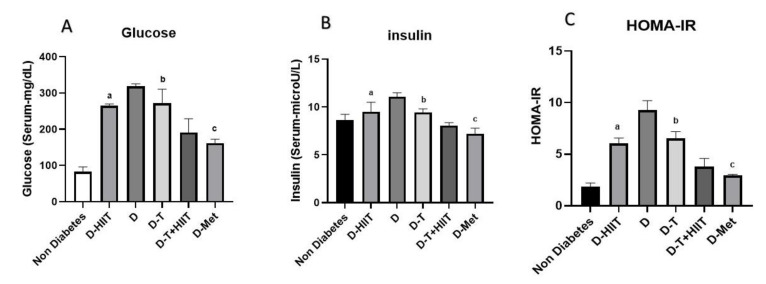
Serum glucose, insulin levels, and HOMA-IR in all groups. (**A**) Serum glucose. (**B**) Serum insulin. (**C**) HOMA-IR. a, b, and c represent the significant difference between the same indicated samples for HIIT, TRF, and metformin effects, respectively, calculated by two-way ANOVA. All data are shown as mean ± SD, *n* = 6 per group. D, diabetes; HIIT, high-intensity interval training; T, time-restricted feeding, Met, metformin.

At the beginning of the induction period, all rats exhibited normal blood glucose levels. FBG levels in the T2D-control group (D) showed a significant increase compared to the non-diabetic control group (non-diabetes) (*p* < 0.001). The exercise groups (D-HIIT and D-T+HIIT) exhibited significant reductions in FBG compared to the non-exercised groups (*p* < 0.001). The D-MET group had significantly lower insulin levels compared to the D group. Rats treated with TRF (D-T and D-T+HIIT) also showed significantly reduced FBG levels compared to the non-TRF (D-HIIT, D, and D-MET) groups (*p* < 0.001). However, there were no significant interactions between HIIT and TRF (*p* = 0.34, Table 3, Figure 3A).

Regarding insulin levels, the D group showed a significant increase in insulin levels compared to the non-diabetes group (*p* < 0.001). Exercised rats (D-HIIT, and D-T+HIIT) demonstrated significantly lower insulin levels compared to the non-exercised groups (*p* = 0.004), while the TRF groups (D-T and D-T+HIIT) also had significantly reduced insulin levels compared to the non-TRF groups (*p* < 0.001). No significant interactions between exercise and TRF were observed (*p* = 0.74, Table 3, Figure 3B).

For IR as determined by HOMA-IR, the D group exhibited significantly higher HOMA-IR levels compared to the non-diabetes group (*p* < 0.001). Rats in the D-MET group also had significantly reduced HOMA-IR compared to the D group (*p* < 0.001). Rats in the exercise groups (D-HIIT and D-T+HIIT) showed significant improvements in HOMA-IR compared to the non-exercised groups (*p* < 0.001). Likewise, TRF-treated rats (D-T and D-T+HIIT) exhibited significantly lower HOMA-IR than the non-TRF groups (*p* < 0.001). There were no significant interactions between exercise and TRF in HOMA-IR levels (*p* = 0.52, Table 3, Figure 3C).

### 3.3. Expressions of IGF-1, AKT, mTOR

**Figure 4:**
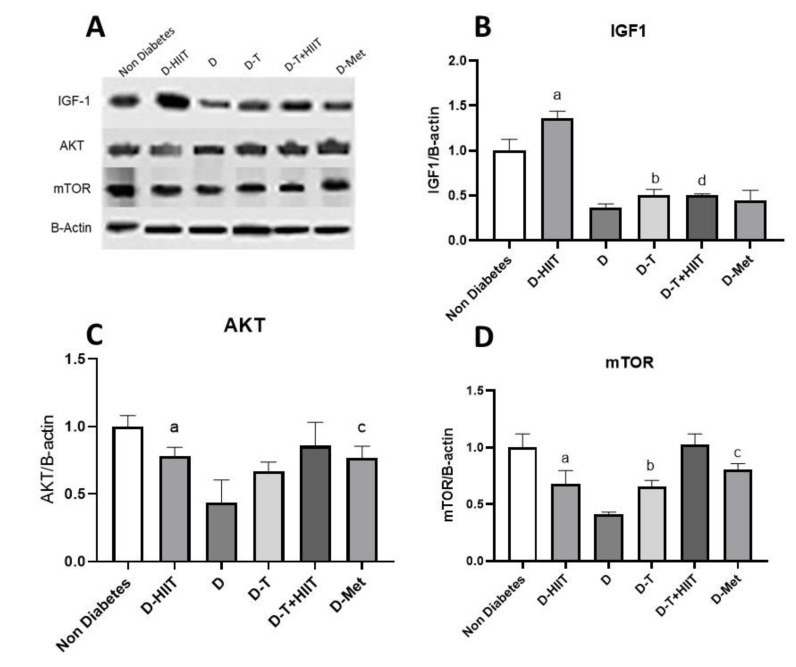
The effects of HIIT, TRF, and metformin on the muscle signaling pathway AKT, FOXO1, PEPCK, Western blotting relative to β-Actin. (**A**) Western blot analysis of the proteins. (**B**) Western blot analysis of muscle tissue IGF-1 levels in type T2D rats. (**C**) Western blot analysis of muscle AKT levels in T2D rats. (**D**) Western blot analysis of muscle tissue mTOR levels in T2D rats. a, b, c, and d represent the significant difference between the same indicated samples for HIIT, TRF, metformin, and combined HIIT and TRF effects, respectively, calculated by two-way ANOVA. All data are shown as mean ± SD, *n* = 6 per group. AKT, protein kinase B; IGF-1, insulin-like growth factor 1; mTOR, mechanistic target of rapamycin; D, diabetes; HIIT, high-intensity interval training; T, time-restricted feeding, Met, metformin.

The protein IGF-1 was significantly (*p* < 0.001) decreased in diabetic rats (D group) compared to non-diabetes group rats and was not significantly changed (*p* = 0.82) in D-MET group rats compared to diabetic rats. There was a significant increase in the content of the protein IGF-1 in the muscle tissue of the D-HIIT group rats compared to the non-exercised groups (Effect size = 0.98, *p* > 0.001, exercise effect), and there was a significant decrease in the rats treated with D-T group compared to the non- TRF groups (effect size = 0.54, *p* = 0.01, TRF effect). Additionally, there was significantly an interaction between exercise and TRF (effect size = 0.96, *p* < 0.001, exercise and TRF combined effect, Table 3, Figure 4A,B).

The protein AKT was significantly (*p* < 0.001) decreased in diabetic rats (D group) compared to non-diabetes group rats, and was significantly (*p* = 0.03) increased in D-MET group rats compared to diabetic rats. There was a significant increase in the content of the protein AKT in the muscle tissue of the D-HIIT group rats compared to the non-diabetes groups (effect size = 0.57, *p* = 0.01, exercise effect), and there was no significant effect in rats treated with TRF compared to the non-TRF groups (effect size = 0.38, *p* = 0.05, TRF effect). Additionally, there were no interactions between exercise and TRF (*p* = 0.32, exercise and TRF combined effect, Table 3, Figure 4A,C).

The protein mTOR was significantly (*p* < 0.001) decreased in diabetic rats compared to the non-diabetes group rats, was significantly (*p* = 0.001) increased in diabetic rats treated with metformin compared to diabetic rats. There was a significant increase in the content of the protein mTOR in the muscle tissue of the exercised rats compared to the non-exercised groups (Effect size = 0.67, *p* = 0.004, exercise effect), and there was a significant increase in the rats treated with TRF compared to the non-TRF groups (effect size = 0.63, *p* = 0.006, TRF effect). Additionally, there were no significant interactions between exercise and TRF (*p* = 0.33, exercise and TRF effect, Table 3, Figure 4A,D).

### 3.4. Histological Changes of the Muscle

**Figure 5:**
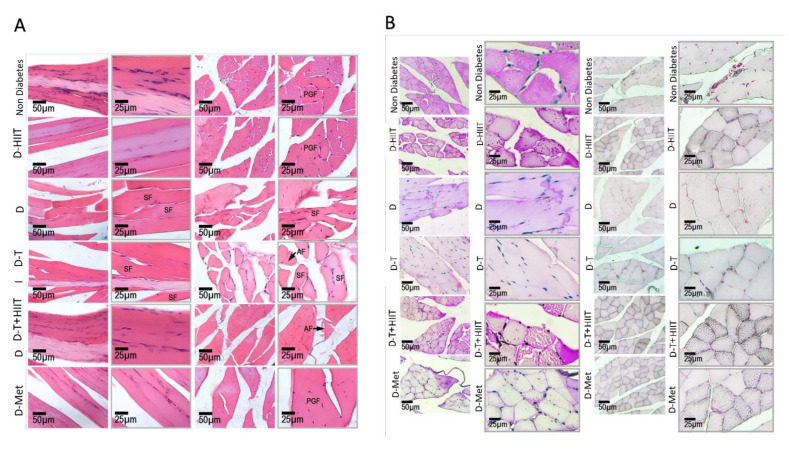
H&E staining and low- and high-magnification photomicrographs for Periodic Acid Schiff (PAS) (**A**) H&E staining of the myofibrils in different groups (Longitudinal and cross-sections): Note normal myofibrils and endomysium connective tissue in the non-diabetes rats with normal distributions of polygonal myofibrils (PGF) in the cross-sections. Black arrow indicates the region of atrophied (AF) myofibrils. The sections from D group are representing atrophied (AF) and shrunken (SF) myofibrils in the Longitudinal and cross-sections, which are ameliorated in the D-HITT, D-T+HIIT, and D-Met groups. (**B**) Periodic Acid Schiff (PAS) and Sudan-Black B staining: the intracytoplasmic carbohydrate (ICC) and the intracytoplasmic lipid/fatty acid (L/FA) are represented in red and black reactions, respectively. Note decreased ICC and diminished L/FA in the D group, which are ameliorated in D-HIIT, D-T+HIIT and at lower degree in the D-Met groups.

The longitudinal and cross-sections of the myofibrils in non-diabetes group exhibited normal endomysium and myofibrils orientation and polygonal myofibrils (PGF) with normal nuclei distribution. However, the sections from the diabetes group showed massive myofibrils atrophy and shrunken myofibrils with increased nuclei distribution versus the non-diabetes rats. This situation was ameliorated in the D-T+HIIT, D-HIIT, and D-Met groups compared to the D group (Figure 5A).

In order to assess the intracytoplasmic carbohydrate (ICC), the PAS staining was considered and the red-stained reactions were considered as ICC. To assess the changes, the software analyses was considered. However, the diabetes significantly decreased the ICC versus non-diabetes group. The mean pixel-based intensity of red-stained reactions (representing ICC) was significantly decreased in the D group. This situation was not significantly changed in the D-TRF group while the ICC was remarkably increased in the D-T+HIIT and the D-HIIT group versus the D group (Figure 5B).

To assess the L/FA droplets/storage, the Sudan-Black B staining was considered and the software analyses were considered similar to PAS staining. The intracytoplasmic L/FA was significantly decreased in the D group, which was ameliorated in the D-HIIT, D-T+HIIT, and the D-Met group (Figure 5B).

## 4. Discussion

The aim of this study was to explore the effects of HFD-induced T2D in rats and the potential therapeutic benefits of HIIT, TRF, and metformin treatment on glucose metabolism, insulin sensitivity, and muscle AKT-IGF-1-mTOR signaling pathways. The findings revealed that both HIIT and TRF significantly improved FBG levels and insulin sensitivity in the T2D-induced rats compared to the diabetes-untreated control (D group). Notably, the HIIT groups (D-HIIT and D-T+HIIT) showed significant reductions in FBG, as well as improvements in insulin and HOMA-IR levels. Furthermore, significant changes were observed in key muscle signaling proteins, with HIIT and TRF interventions leading to increased expression of IGF-1, AKT, and mTOR in muscle tissue, while the D group exhibited decreased levels. Histological analysis indicated that HIIT and TRF helped mitigate muscle atrophy and improved myofibril structure, while also enhancing intramyocellular lipid storage. Although both HIIT and TRF independently improved insulin sensitivity and protein signaling, their combination provided additive benefits, particularly evident in IGF-1 expression and muscle morphology.

While previous studies have established the detrimental effects of HFD and IR on insulin signaling pathways, our results extend these findings by showing that combining HIIT and TRF partially restores AKT and mTOR expression, suggesting improved muscle anabolic potential. In the current study, we investigated the effects of HIIT and TRF on the AKT-IGF1-mTOR signaling pathway in the muscle tissue of T2D-induced rats before and after HIIT and TRF administration. Moreover, we compared the results with those T2D-induced rats which received MET. We used HOMA-IR to evaluate IR and the quantitative insulin sensitivity check index (QUICKI) for insulin sensitivity. In this study, we studied the methods of H&E staining and PAS to examine the muscle tissue. T2D was induced by injecting low-dose STZ into the rats fed with an HFD. Our findings showed that treatment with exercise and fasting improves FBG, IPGTT, IR, IGF-1, AKT, and mTOR protein content in diabetic rats.

For many years, researchers have identified a strong association between obesity, of which obesity accomplished with T2D-induced IR is often cited as the primary mechanism underlying this association [38]. Obesity is known to impair the skeletal muscle’s ability to regulate glucose uptake in response to insulin, which is a critical factor in the development of IR [39]. In line with this issue, it has been reported that obesity enhances fat accumulation within the skeletal muscle and is correlated with the incidence of IR [39]. In addition, an increased IR in T2D patients has been shown to be due to increased intracellular lipid content, which in turn leads to impaired insulin signaling and insulin action in skeletal muscle [40]. Our study’s findings demonstrated that HFD and STZ led to elevated FBG, which is consistent with previous studies [41,42]. Previous research highlighted that HFD contributes to increased body weight, a steady rise in blood glucose levels, and a gradual increase in hyperinsulinemia, all of which indicate a progressive worsening of IR [43,44]. In accordance with this, the PAS staining of muscle tissue revealed that HIIT and TRF significantly elevated intracellular glucose levels, indirectly supporting the notion that HIIT and TRF could help reverse T2D-induced IR in skeletal muscle. However, we failed to show an increased lipid storage in the muscle samples.

To understand this subject, one should note that exercise and fasting are two effective non-medical interventions that have been shown to improve insulin sensitivity in diabetic rats [45]. The cellular mechanisms underlying this effect has been shown to have a profound impact on IR and insulin signaling pathway in rat skeletal muscle, which is a crucial aspect of glucose metabolism [46,47]. In line, they modulate IRS-1 phosphorylation, GLUT4 expression, promoting muscle fiber type switching, reducing inflammation, and improving mitochondrial function, PI3K/AKT signaling, AMPK activity, and mTORC1 inhibition [48,49]. These findings have important implications for the prevention and treatment of metabolic disorders characterized by IR by considering exercise and fasting [50,51]. In addition to considering the aforementioned parameters and PAS staining, we found that the combination of HIIT and TRF positively affected insulin sensitivity.

Building on these findings, our results suggest that TRF alone did not significantly improve AKT expression, yet its influence on mTOR and IGF-1 was more pronounced. This suggests that TRF may exert its benefits through nutrient-sensing pathways, potentially through circadian-aligned feeding patterns and fasting-induced AMPK activation. However, TRF did not show interaction effects with HIIT in the two-way ANOVA, indicating that its benefits might be complementary rather than synergistic. Accordingly, the AUC in IPGTT of male diabetic rats was increased compared to the control group. Although the D-T+HIIT group showed the most consistent improvements across metabolic and molecular markers, the absence of statistically significant interaction effects for key variables like AKT and mTOR suggests that these effects may be additive rather than synergistic. Therefore, future studies should consider designs powered specifically to test interaction effects over time.

Conversely, the HIIT ameliorated the glucose tolerance and reduced the peak glucose IPGTT. This condition may be attributed to the increased insulin levels resulting from HIIT and TRF, clearly demonstrating that the HIIT and fasting regimen used in this study are able to induce significant effects.

Impaired muscle glycogen synthesis is the main deficiency responsible for IR in the early phases of the progression of T2D. In the physiological condition, insulin through facilitating the glucose transport into the cytoplasm, maintains the PI3-K/Akt-related metabolic signaling in skeletal muscle [52]. Insulin binds to the insulin receptor in skeletal muscle, triggering the phosphorylation of three key tyrosine molecules. This process activates IRS1, which moves to the cell membrane, is phosphorylated, and ultimately leads to the activation of PI3-K and downstream AKT. Next, Akt phosphorylates AS160 immediately, transferring glucose into the skeletal muscle [53]. Thus, the IRS-1/PI3-K/AKT pathway is necessary for glucose uptake in skeletal muscles [54] and any disruption in this signaling is able to disrupt the glucose up-taking machinery in muscles. This has been shown by prior studies showing that AKT or IRS adaptor protein knockout or knockdown clearly reduces insulin-induced glucose uptake, whereas AKT overexpression improves glucose uptake [55]. Considering these factors, we examined the expression levels of AKT under different conditions. Our findings indicate that, similar to MET, both HIIT and TRF (mainly in combined form) significantly up-regulate AKT protein expression, which is reduced in T2D. Therefore, we suggest that HIIT and TRF, at least partially, help muscular cells overcome T2D-induced IR by regulating AKT expression.

The AKT3-mTOR-IGF-1 signaling pathway plays a crucial role in regulating glucose uptake in muscle cells, acting as a key mechanism for insulin sensitivity and muscle metabolism [56]. Upon insulin binding to its receptor, AKT3 is activated, which then initiates downstream signaling through mTOR and IGF-1 [57]. mTOR, a central regulator of cell growth and metabolism, enhances protein synthesis and cellular processes necessary for muscle function [58], while IGF-1 stimulates anabolic activity, promoting muscle repair and growth [7]. Earlier studies have shown that disruptions in this pathway, often seen in conditions like T2D, lead to impaired glucose uptake and IR in muscle cells [59,60]. These findings underscore the importance of the AKT3-mTOR-IGF-1 pathway in maintaining muscle glucose homeostasis and highlight potential therapeutic targets for managing IR in metabolic diseases.

To investigate the effects of T2D, HIIT, TRF, and MET on this pathway, we analyzed the mTOR and IGF-1 protein expression across all experimental groups. Our results demonstrated that HIIT significantly increased IGF-1 expression compared to the untreated group. Additionally, HIIT, TRF, and MET all significantly up-regulated mTOR expression in comparison to the untreated T2D-induced rats (D group). Considering these findings, we propose that HIIT, by modulating both IGF-1 and mTOR expression, and TRF, through the up-regulation of mTOR expression, effectively support the AKT3-IGF-1-mTOR pathway in maintaining muscle glucose homeostasis. These results emphasize the potential of HIIT and TRF (mainly in combined form) as therapeutic strategies for managing IR in T2D condition.

Regarding the effect of metformin on IGF-1 and mTOR expression, it is important to note that while metformin has been shown in previous studies to increase IGF-1 levels and improve muscle function [52,53], our findings did not show a statistically significant change in IGF-1 expression in the MET group compared to diabetic controls. However, metformin significantly increased mTOR expression, and previous evidence also suggests that it enhances AKT phosphorylation, which may contribute to improved glucose utilization and insulin sensitivity [61]. While metformin slightly improved AKT expression, its effects on IGF-1 were less pronounced compared to HIIT. These results may reflect tissue-specific or dose-dependent responses, as metformin’s influence on IGF-1 appears inconsistent across studies.

This study highlights the beneficial effects of HIIT, TRF, and metformin on glucose metabolism, insulin sensitivity, and muscle AKT3-IGF-1-mTOR signaling pathways in a T2D rat model. The findings demonstrated that both HIIT and TRF significantly improved FBG levels and insulin sensitivity compared to untreated diabetic rats, with HIIT exerting more pronounced effects on muscle IGF-1 and mTOR expression. Histological analysis further revealed that HIIT and TRF mitigated muscle atrophy and improved muscle fiber structure, supporting the notion that these interventions can help reverse T2D-induced IR. Additionally, the study emphasized the critical role of the AKT3-IGF-1-mTOR pathway in regulating glucose uptake and insulin sensitivity in skeletal muscle, with HIIT and TRF contributing to the activation of this pathway, thus improving muscle glucose homeostasis. The results also suggested that metformin, while effective in increasing IGF-1 and modulating mTOR expression, showed less impact than HIIT in regulating these key proteins. Overall, these findings underscore the potential of HIIT and TRF as effective therapeutic strategies for managing IR and improving metabolic health in T2D. Further studies are needed to explore the long-term effects and underlying mechanisms of these interventions, which could offer promising alternatives to conventional pharmacological treatments for metabolic diseases.

According to the results of our study, the combination of exercise and fasting has improved the protein content of AKT, IGF1, and mTOR. In this regard, it is expected that fasting decreases the concentration of mTOR protein and increases the protein content of AKT, IGF-1, and the exercise performed in the fasting state may accelerate the effect of fasting on proteins. The change in the content of these proteins due to exercise and fasting can also affect the metabolism and improvement of diabetic blood glucose. It has been reported that the activation of several proteins and transcription factors (as “master regulators” of metabolism) can induce metabolic changes during fasting and exercise [62]. Decreased intracellular concentration of AKT is one of the things that is related to IR. Exercise and fasting appear to have a synergistic relationship in modulating cellular signaling pathways, particularly those involved in cell metabolism and growth. One key intersection is the AKT-mTOR-IGF-1 axis. Exercise triggers the activation of adenosine monophosphate-activated protein kinase (AMPK), which phosphorylates and inhibits mTOR, leading to reduced protein synthesis and increased autophagy. This reduces the need for IGF-1, allowing for a decrease in its production and promoting cellular cleaning and renewal. Fasting, which also inhibits mTOR through the activation of AMPK, further enhances this effect. The combination of exercise and intermittent fasting can lead to a more pronounced inhibition of mTOR, resulting in increased autophagy and improved cellular stress resistance. It is important to note that mTOR is contextually regulated chronic fasting and typically inhibits mTOR to promote autophagy, whereas acute exercise induces transient mTOR activation to stimulate muscle repair and protein synthesis. Our observed increase in mTOR likely reflects this acute anabolic adaptation.

As a result, the cellular environment becomes more primed for cellular renewal, growth, and repair, while also reducing the risk of disease associated with uncontrolled cell growth and proliferation [63].

Our study showed that combining HIIT and TRF can help improve muscle glycogen stores and increase the body’s capacity to use glucose. Under the influence of HIIT, glycogen stores are increased and glucose utilization in muscles is improved. In addition, intermittent fasting allows the body to use stored fat to produce glucose in times of energy deficiency, preserving glycogen stores for times when it is needed. The inability to restore muscle glycogen levels during the fed state may be explained by the increased state of IR induced by HFD, which could be caused by low-grade inflammation, accumulation of intramuscular lipid metabolites, such as fatty acid-induced complications. Indeed, other models of IR obese rats confirm that skeletal muscle glycogen decreases during the recovery period [16,64], which is consistent with the results obtained from our study. Acute exercise is known to improve glucose uptake and glycogen synthesis, even in IR individuals [65].

Our results showed that metformin can help reduce IR and improve glucose absorption by affecting the content of these proteins. Studies have shown that metformin can increase the level of IGF-1, which helps improve protein synthesis and muscle function. In addition, metformin helps to reduce inflammation and improve metabolic status by reducing mTOR activity [66,67]. A study showed that metformin increases the phosphorylation of AKT in skeletal muscles, which leads to improved glucose utilization and lower blood glucose levels [68].

Another study showed that metformin did not improve the positive effects of exercise on performance, although it protected myocytes against exercise-induced injury. In the study of the role of metformin in osteoblast differentiation in T2D, it was shown that the administration of metformin decreases the serum levels of IGF-1 [69,70]. Further research should assess the long-term implications of combining HIIT and TRF, including muscle protein turnover, mitochondrial biogenesis, and glucose transporter activity. Additionally, since our study used male rats, future studies should explore potential sex-based differences in response to these interventions. It would also be valuable to assess time-course changes in AKT, mTOR, and IGF-1 expression during different stages of intervention (e.g., 2, 6, 10 weeks) to understand temporal dynamics.

One of the main limitations that affected the design and conduct of the study was financial constraints. Due to financial constraints, it was not possible to measure and examine some additional metabolic factors that could provide useful information about overall metabolic status. These factors could have included blood lipid profiles, inflammatory markers, various hormone levels, and other important metabolic parameters that could have contributed to a more accurate assessment of metabolic health status. In addition to financial constraints, there were other factors such as time constraints and limited access to sophisticated technologies.

## 5. Conclusions

HIIT and TRF are two dietary interventions that have been shown to improve insulin sensitivity and glucose metabolism in various studies. This study found that the combination of HIIT and TRF resulted in a significant improvement in muscle glucose uptake and storage in T2D rats. This was achieved through activation of the AKT-IGF-1-mTOR signaling pathway, which is a key mechanism regulating glucose metabolism in muscle tissues. In addition, this study showed that HIIT and TRF individually activated certain components of the AKT-IGF-1-mTOR pathway, but their combined effect resulted in a more robust activation of the entire pathway. Our results suggest that the combination of HIIT and TRF may exert additive effects on improving glucose metabolism in T2D rats, though further studies are needed to confirm potential synergy. Therefore, we conclude that the combination of HIIT and TRF may be a promising therapeutic approach for improving insulin sensitivity and glucose metabolism in T2D patients. These findings support further translational studies to test the clinical relevance of HIIT and TRF in individuals with T2D.

## Figures and Tables

**Table 1:** Diet Composition.

High-Fat Diet (60%)	Standard Diet	Parameters
24	23	Protein (%)
26	50.3	Carbohydrate (%)
35	4.5	Fat (%)
15	22.2	Others (%)
60	10	Fat (%)
5.2	3.1	Calories (kcal/g)

**Table 2:** High-intensity interval training protocol.

Week	Warm-Up (Min)	Intensity Warm-Up (%Vo2max)	Treadmill Speed Warmup Time (Meters/Min)	Repetition	Ratio of Training Time to Rest (Min)	Training Intensity (%Vo2max)	Treadmill Speed During Exercise (Meters/Min)	Rest Time Intensity (%Vo2max)	Treadmill Speed at Rest (Meters/Min)	Cool Down (Min)	Intensity Cool Down (%Vo2max)	Treadmill Speed Cool Down Time (Meters/Min)	Total Duration (Min)
							Vmax test evaluation						
1	5	30–40	10	15	3:2	85–90	26	30–40	10	5	30–40	10	48
2	5	30–40	10	15	3:2	85–90	26	30–40	10	5	30–40	10	48
3	5	30–40	10	15	3:2	85–90	26	30–40	10	5	30–40	10	48
4	5	30–40	10	15	3:2	85–90	26	30–40	10	5	30–40	10	48
							Vmax test evaluation						
5	5	30–40	12	15	4:2	85–90	30	30–40	12	5	30–40	12	56
6	5	30–40	12	15	4:2	85–90	30	30–40	12	5	30–40	12	56
7	5	30–40	12	15	4:2	85–90	30	30–40	12	5	30–40	12	56
8	5	30–40	12	15	4:2	85–90	30	30–40	12	5	30–40	12	56
9	5	30–40	12	15	4:2	85–90	30	30–40	12	5	30–40	12	56
10	5	30–40	12	15	4:2	85–90	30	30–40	12	5	30–40	12	56

**Table 3:** Two-way ANOVA.

Two-Way ANOVA for Protein Content of AKT, IGF1, and mTOR						
	Main Effect, *p*-Value, Effect Size				Interaction, *p*-Value, Effect Size	
Parameters	HIIT		TRF		TRF × HIIT	
	*p*-Value	Effect Size	*p*-Value	Effect Size	*p*-Value	Effect Size
**Serum glucose (mg/dL)**	<0.001	0.66	<0.001	0.61	0.34	0.075
**Serum insulin** **(microU/L)**	<0.001	0.67	<0.001	0.68	0.74	0.009
**HOMA-IR**	<0.001	0.87	<0.001	0.63	0.52	0.035
**IGF-1/β-Actin protein**	<0.001	0.98	0.015	0.54	<0.001	0.96
**AKT/β-Actin protein**	0.011	0.57	0.057	0.38	0.32	0.12
**mTOR/β-Actin protein**	0.004	0.67	0.006	0.63	0.33	0.116

## Data Availability

The data that support the findings of this study are available from the corresponding author upon reasonable request. They are not publicly available due to privacy and legal restrictions.
